# Prioritization of genes involved in endothelial cell apoptosis by their implication in lymphedema using an analysis of associative gene networks with ANDSystem

**DOI:** 10.1186/s12920-019-0492-9

**Published:** 2019-03-13

**Authors:** Olga V. Saik, Vadim V. Nimaev, Dilovarkhuja B. Usmonov, Pavel S. Demenkov, Timofey V. Ivanisenko, Inna N. Lavrik, Vladimir A. Ivanisenko

**Affiliations:** 1 Laboratory of Computer-Assisted Proteomics, Institute of Cytology and Genetics, Siberian Branch, Russian Academy of Sciences, Prospekt Lavrentyeva 10, Novosibirsk, 630090 Russia; 2 Laboratory of Surgical Lymphology and Lymphodetoxication, Research Institute of Clinical and Experimental Lymрhology – Branch of the Institute of Cytology and Genetics, Siberian Branch of Russian Academy of Sciences, st. Timakova 2, Novosibirsk, 630117 Russia; 3 Novosibirsk State University, st. Pirogova 1, Novosibirsk, 630090 Russia; 4 Department of Neurosurgery, Ya. L. Tsivyan Novosibirsk Research Institute of Traumatology and Orthopedics, Ministry of Health of the Russian Federation, st. Frunze 17, Novosibirsk, 630091 Russia; 5 Translational Inflammation Research, Institute of Experimental Internal Medicine, Otto von Guericke University Magdeburg, Medical Faculty, Pfalzer Platz 28, 39106 Magdeburg, Germany

**Keywords:** Lymphedema, Endothelial cell apoptosis, ANDSystem, Associative gene networks, Gene prioritization

## Abstract

**Background:**

Currently, more than 150 million people worldwide suffer from lymphedema. It is a chronic progressive disease characterized by high-protein edema of various parts of the body due to defects in lymphatic drainage. Molecular-genetic mechanisms of the disease are still poorly understood. Beginning of a clinical manifestation of primary lymphedema in middle age and the development of secondary lymphedema after treatment of breast cancer can be genetically determined. Disruption of endothelial cell apoptosis can be considered as one of the factors contributing to the development of lymphedema. However, a study of the relationship between genes associated with lymphedema and genes involved in endothelial apoptosis, in the associative gene network was not previously conducted.

**Methods:**

In the current work, we used well-known methods (ToppGene and Endeavour), as well as methods previously developed by us, to prioritize genes involved in endothelial apoptosis and to find potential participants of molecular-genetic mechanisms of lymphedema among them. Original methods of prioritization took into account the overrepresented Gene Ontology biological processes, the centrality of vertices in the associative gene network, describing the interactions of endothelial apoptosis genes with genes associated with lymphedema, and the association of the analyzed genes with diseases that are comorbid to lymphedema.

**Results:**

An assessment of the quality of prioritization was performed using criteria, which involved an analysis of the enrichment of the top-most priority genes by genes, which are known to have simultaneous interactions with lymphedema and endothelial cell apoptosis, as well as by genes differentially expressed in murine model of lymphedema. In particular, among genes involved in endothelial apoptosis, *KDR, TNF, TEK, BMPR2, SERPINE1, IL10, CD40LG, CCL2, FASLG* and *ABL1* had the highest priority. The identified priority genes can be considered as candidates for genotyping in the studies involving the search for associations with lymphedema.

**Conclusions:**

Analysis of interactions of these genes in the associative gene network of lymphedema can improve understanding of mechanisms of interaction between endothelial apoptosis and lymphangiogenesis, and shed light on the role of disturbance of these processes in the development of edema, chronic inflammation and connective tissue transformation during the progression of the disease.

**Electronic supplementary material:**

The online version of this article (10.1186/s12920-019-0492-9) contains supplementary material, which is available to authorized users.

## Background

Lymphedema is a chronic progressive disease, resulting in a significant loss of productivity, which affects more than 150 million people worldwide [1]. At the heart of the pathogenesis of lymphedema lies the appearance of chronic high-protein edema, characterized by the pathological accumulation of intercellular fluid with large-molecule proteins in the interstitial space due to a defect in lymphatic drainage caused by congenital malformation (primary lymphedema), lymphatic obstruction or by the destruction of lymphatic vessels (secondary lymphedema). Long existence of the high-protein edema causes chronic inflammation leading to the replacement of adipose tissue with connective tissue, increasing the volume of connective tissue matrix, which subsequently leads not only to an increase in size of body parts, but also to a secondary disturbance of lymphatic transport and drainage [2]. Most often it affects the lower extremities, but can also affect the upper limbs, face, trunk, external genitalia, etc. [3]. Primary lymphedema is a disease caused by dysfunction of lymphatic vessels, their aplasia, dysplasia and hypoplasia. Disturbance of drainage function of lymphatic vessels leads to accumulation of fluid rich in proteins under the epidermis. Most often, edema is localized on the lower limbs. Family forms of primary lymphedema are most often an autosomal dominant disease, but autosomal recessive types of inheritance of this pathology are also found [4]. It is believed that developmental defects and the resulting dysfunction of the lymphatic system are the cause of primary lymphedema, as well as associated syndromes, and can be genetically determined [5]. Secondary lymphedema is most often the result of a complex treatment of breast cancer, including removal of the axillary lymph nodes and/or radiation therapy [6]. Recently a number of associations between gene polymorphisms, including cytokine genes [7], and the development of breast cancer–related lymphedema were identified [8, 9]. One of the possible causes of clinical manifestation of primary lymphedema in middle age and the development of secondary lymphedema after treatment of breast cancer may be apoptosis of the endothelium, whose role is discussed in publications on the pathogenesis of lymphedema [10, 11].

Apoptosis is a form of cell death, characterized by a number of morphological and molecular features, including exposure of phosphatidylserine on the cell membrane, blebbing of the plasma membrane, cell shrinkage, cytoskeleton rearrangement, nuclear collapse, chromatin condensation, DNA fragmentation and formation of “apoptotic bodies” [12]. A number of works discuss the role of endothelial cell apoptosis in the pathogenesis of lymphedema [10, 11, 13, 14]. However, the association between genes involved in endothelial apoptosis and genes associated with lymphedema has not been yet analyzed.

One of the most effective and frequently used approaches for the identification of the potential associations between genes and diseases is a prioritization [15]. Nowadays, there are many known methods for the prioritization of genes. A first, and the largest group of such methods is based on the analysis of genomic and transcriptomic data, as well as data on homologues [16]. The second class of prioritization methods involves approaches based on the analysis of gene networks and protein-protein interactions networks. An example of such methods is GUILD [17]. Integrated methods that combine approaches from the first and the second groups represent the third group. The well-known examples of such systems from the third group are ToppGene [15] and Endeavour [18].

Previously, we developed criteria for the prioritization of genes, by using the well-known systems and also considering the structure of associative gene networks from ANDSystem [19, 20]. ANDSystem is a computer tool, designed for the automated extraction of knowledge from the texts of scientific publications and automatic reconstruction of the associative gene networks by using the retrieved information, describing the mechanisms of diseases, as well as other complex phenotypic traits. The knowledge base of ANDSystem contains over 30 million facts describing genetic regulation, gene associations with diseases, protein-protein interactions, catalytic reactions, transport pathways, etc., extracted from more than 25 million PubMed abstracts [21, 22]. In particular, ANDSystem was used for the identification of candidate genes associated with comorbidity of preeclampsia, diabetes and obesity [23], asthma and tuberculosis [24], as well as asthma and hypertension [19].

In this study, our prioritization criteria were applied to identify the endothelial apoptosis-related genes, potentially involved in lymphedema. Three approaches were used to assess the quality of prioritization, based on the evaluation of the enrichment of the list of top priority genes by genes for which there was indirect evidence confirming their association with lymphedema: (1) enrichment with genes shared between lymphedema and endothelial apoptosis; (2) enrichment with genes, the names of which are significantly co-occurring in full-text articles with the key word «lymphedema»; (3) enrichment with genes differentially expressed in the murine model of lymphedema. All these quality criteria showed significant enrichment.

Among the genes with the highest priority *TNF, TEK, BMPR2, SERPINE1, IL10, CD40LG, CCL2, FASLG* and *ABL1* can be distinguished. These genes can be used to plan experiments confirming their association with lymphedema, and to be further considered as new candidates for genotyping. The found genes can become the basis for a better understanding of the mechanisms of interaction between endothelial apoptosis and lymphedema.

## Methods

The list of genes associated with lymphedema (Additional file 1: Table S1) was extracted from the CTD [25], Malacards [26], KEGG [27], HPO [28] and DisGeNET [29] databases, available for January 2018. This list was additionally expanded with genes associated with lymphedema, extracted from ANDSystem [30].

The over-represented Gene Ontology (GO) biological processes were identified using the DAVID 6.8 tool [31] with the following parameters: the organism – «Homo sapiens», Gene_Ontology – «GOTERM_BP_DIRECT».

Reconstruction of associative gene networks was carried out by using the ANDSystem [21, 22].

The betweenness centrality of a node in a gene network was estimated using the networkx package of the Python programming language [32]. This indicator characterizes the number of shortest pathways between all pairs of vertices of analyzed graph passing through a given vertex and reflects the functional significance of gene in gene network.

The Mann-Whitney criterion was calculated using the mannwhitneyu function of the scipy.stats package of the Python programming language [33].

The list of human genes involved in the Gene Ontology biological process «apoptotic process» was obtained using the AmiGO system [34] by the «GO:0006915» query and Organism filter set to «Homo sapiens».

The list of human genes involved in the Gene Ontology biological process «endothelial cell apoptotic process» was obtained with AmiGO using the «GO:0072577» query and with Organism filter set to «Homo sapiens».

**Fig. 1:**
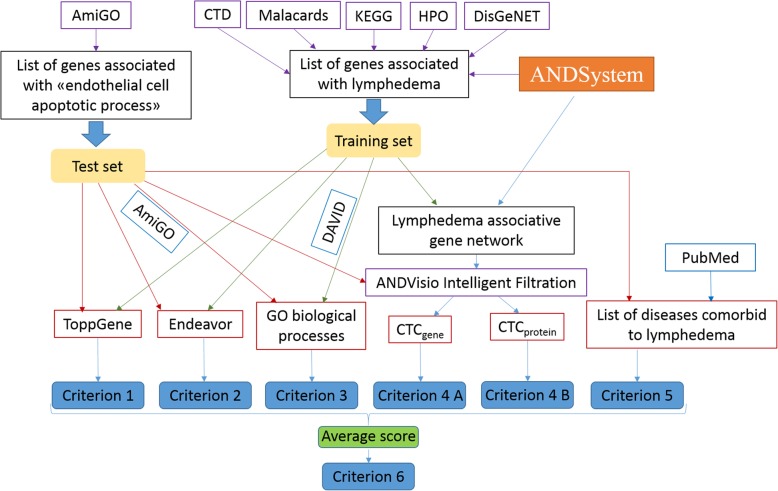
The prioritization pipeline overview

For the gene prioritization six criteria were used (Fig. 1), which were discussed in our previous studies devoted to the analysis of asthma, hypertension and Parkinson’s disease [19, 20].

Criteria 1 and 2 were the well-known prioritization methods: ToppGene [15] and Endeavour version 3.71 [18], respectively. These resources allow to perform ranking of a test set of genes by a training set of genes according to specific criteria characterizing the proximity of genes from the test set to the genes from the training sample. The methods of these resources use the genetic information (co-localization in the genome), the functional properties of genes (participation in the same GO categories), etc., as well as properties of the vertices of the graph of protein-protein networks. A list of genes associated with lymphedema, described above, was used as a training set for each of these methods. As a test set, the list of genes involved in endothelial cell apoptosis, described above, was used. For the ToppGene the “all Feature” parameter was selected in the “Training parameters” section, and the ranking of genes was based on the value of the “Rank” output parameter. In case of the Endeavour system all settings were set to default, the gene ranking was based on “*P*-value”. Thus, the lowest ranks had genes with the lowest “P-value”, while genes with the highest “P-value” obtained the highest ranks.

Criterion 3 was calculated as the proportion of the biological processes, over-represented for a set of genes associated with lymphedema (Additional file 2: Table S2), to all Gene Ontology biological processes where the analyzed gene was involved. Information on the involvement of gene in the Gene Ontology biological process was obtained from the AmiGO system [34]. Ranks for genes were determined with the sorting of the list of genes by descending the proportion of over-represented Gene Ontology biological processes. Thus, the lowest ranks were assigned to genes with the largest proportion of over-represented Gene Ontology biological processes.

Criterion 4 was based on the use of the cross-talk centrality (CTC), calculated using the «Intelligent Filtration» function of ANDSystem. Within this criterion, CTCs were calculated separately for genes from the gene network (criterion 4A) and for their products (criterion 4B). Thus, two indices (CTC_gene_ and CTC_protein_) were determined for each gene. The centrality of CTC_gene_ was calculated using the following formula:

$$ {CTCgene}_i={N}_i/M $$

where *N*_*i*_ – is a number of interactions of the i-th gene with members of the associative gene network of lymphedema; M – is a total number of nodes from the associative gene network of lymphedema. CTC_protein_ was determined for the i-th protein or miRNA in a same way. CTC_gene_ and CTC_protein_ ranks were determined by sorting the values in descending order the same way as it was done for the criterion 3.

Criterion 5 represents the number of diseases comorbid to lymphedema, which are associated with the analyzed gene. Comorbid diseases are considered to be simultaneously present in one patient more often than can be expected for accidental reasons [35, 36]. The list of diseases comorbid to lymphedema was manually created by analyzing the publications corresponding to the following query to the PubMed database: “lymphedema and (comorbid or comorbidity)”. A total of 80 publications were manually analyzed and six comorbid diseases were found (Additional file 3: Table S3). All interactions between the analyzed genes and these six comorbid diseases were established using ANDSystem. In addition, the rank by criterion 5 was determined by sorting the list of genes in descending order of the number of comorbid diseases associated with these genes.

In the cases when several genes had the same value of the criterion by which the ranking was performed, the rank of these genes was calculated similarly to the Spearman rank correlation coefficient [37]. I.e., such genes were assigned with the same rank, equal to the average arithmetic ranks of these genes, according to their position, in the sorted list of genes.

Criterion 6 was calculated as the average value of ranks obtained from the criteria 1–5.

The statistical significance of the co-occurrence of gene names from the test set and lymphedema in full-text articles was estimated as follows:

(1) The number of full-text publications presented in the PubMed Central system (https://www.ncbi.nlm.nih.gov/pmc/) was calculated according to the following parameters:

(a) the official name of the analyzed gene is co-occurring with the “lymphedema” word,

(b) the official name of the analyzed gene is mentioned in the article,

(c) the word “lymphedema” is mentioned in the article.

(2) Using the hypergeometric distribution, implemented in the hypergeom.sf function of the scipy.stats package of the Python programming language [33], the statistical significance of the co-occurrence of names of the analyzed genes and lymphedema was assessed.

(3) Correction for multiple comparison of FDR was carried out using the «Signed Differential Mapping» tool (https://www.sdmproject.com/utilities/?show=FDR) [38].

The correlation between the ranks of criteria 1–6 and the significance of co-occurrence of the analyzed genes names and lymphedema in the full-text articles was carried out using the Spearman’s rank correlation coefficients. The calculations were performed using the Social Science Statistics system (http://www.socscistatistics.com/tests/spearman/default2.aspx).

Data on gene expression in the mouse surgical model of acute lymphedema induction were collected from the GEO resource (https://www.ncbi.nlm.nih.gov/geo/) using the «GSE4333» id [39]. Differentially expressed genes (*p*-value < 0.05) for the following groups: (1) «Lymphedema Tail Skin» (test) versus «Normal Tail Skin» (control 1); and (2) «Lymphedema Tail Skin» versus «Surgical Sham Control Tail Skin» (control 2), were identified using the GEO2R tool (http://www.ncbi.nlm.nih.gov/geo/geo2r/). Mapping of mouse genes on human orthologous genes was carried out using the HomoloGene database (https://www.ncbi.nlm.nih.gov/homologene).

## Results and discussion

### Associative gene network of lymphedema

**Fig. 2:**
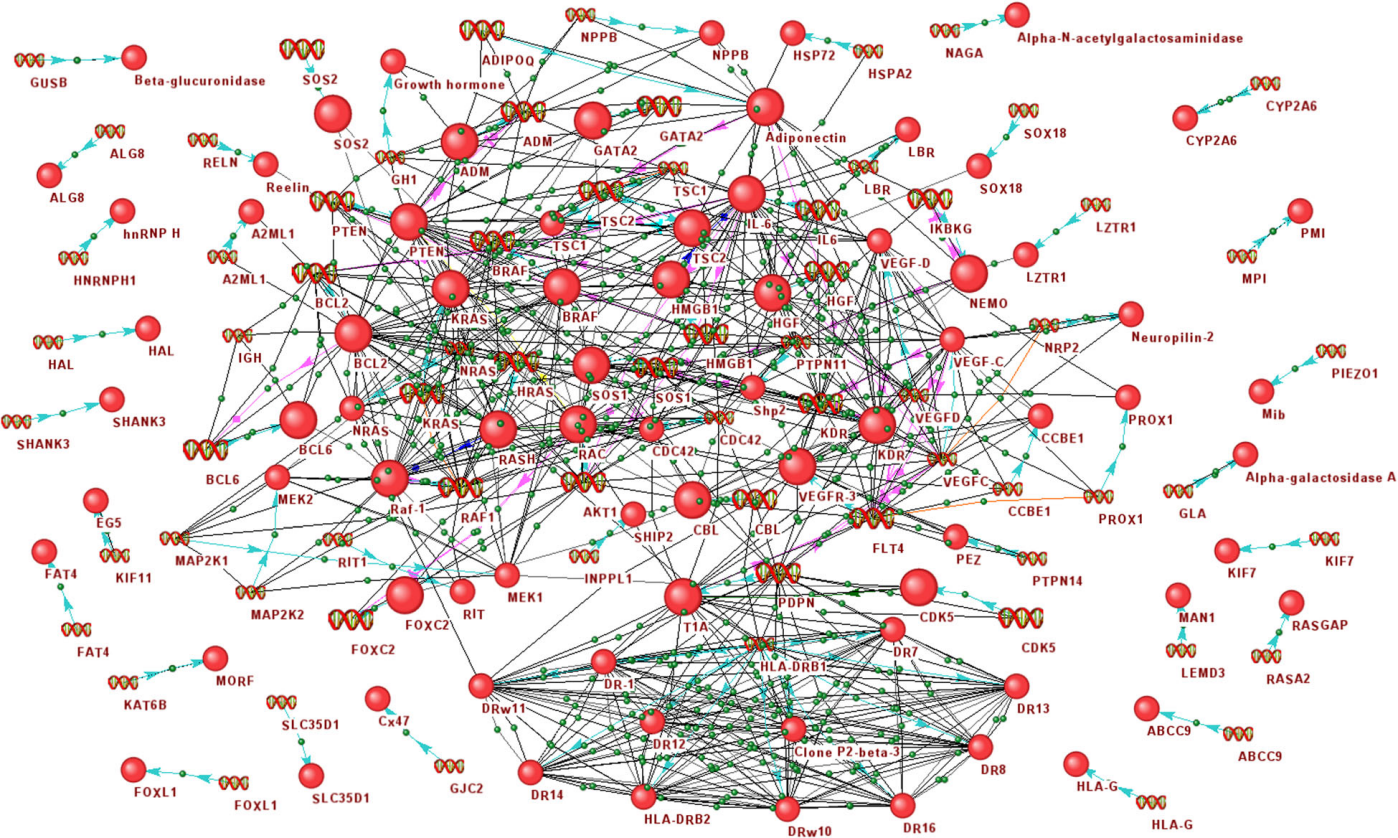
The associative gene network of lymphedema. Genes and proteins involved in the Gene Ontology biological process «apoptotic process» (GO:0006915) are shown with large icons

Analysis of information from the CTD [25], Malacards [26], KEGG [27], HPO [28] and DisGeNET [29] databases and from ANDSystem, revealed 69 genes associated with lymphedema (Additional file 1: Table S1). The obtained list of genes was applied to ANDSystem for the reconstruction of the associative gene network of lymphedema. The reconstructed network was automatically expanded in ANDSystem with the products of these genes. The obtained gene network contained 69 genes and 78 proteins, as well as 709 interactions between them (Fig. 2), including the following interaction types: association (460 interactions), protein-protein interaction (128 interactions), gene expression (78 interactions), regulation of gene expression (30 interactions), co-expression (6 interactions), regulation of transport (2 interactions), regulation of degradation (2 interactions), catalysis (2 interactions) and regulation of activity (1 interaction). Twenty one genes from the network were interacting only with their own products, while the remaining 48 genes and 57 proteins were highly connected with each other (Fig. 2).

Analysis of the overrepresentation of Gene Ontology (GO) biological processes for a set of genes associated with lymphedema revealed 40 processes significantly enriched with these genes (Additional file 2: Table S2). Among the most over-represented processes were lymphangiogenesis, endothelial cell proliferation, ERK1/ERK2 cascade and VEGF signaling pathway. The role of these biological processes in the pathogenesis of lymphedema is actively discussed in the literature [8, 40–42]. For example, Saito et al., 2013 studied the possibility of therapeutic lymphangiogenesis, using hepatocyte growth factor for the treatment of lymphedema [40]. Miaskowski et al., 2013, described the important role of VEGF signaling pathway in lymphangiogenesis associated with inflammation in lymphedema [8].

It should be noted that among the significantly over-represented GO biological processes were also processes of positive and negative regulation of the apoptosis (Additional file 2: Table S2); this is consistent with recent studies suggesting the important role of apoptosis in the pathogenesis of lymphedema [14]. The associative gene network of lymphedema involved 24 genes/proteins from the GO biological process entitled “apoptotic process” (Fig. 2). Analysis of the centrality of nodes from the gene network of lymphedema (Additional file 4: Table S4) showed that the average value of the betweenness centrality for apoptosis genes (equal to 303) statistically significantly exceeds the value calculated for all the nodes of the gene network (equal to 128), with *p*-value< 10^− 6^, according to the Mann-Whitney criterion. The high centrality of the apoptotic genes in the lymphedema gene network can be indirect evidence of the key role of the apoptosis process in the pathogenesis of lymphedema.

### Prioritization of endothelial apoptosis genes by their potential association with lymphedema

Considering the importance of apoptosis of endothelial cells in the pathogenesis of lymphedema [10, 11, 13, 14], we performed a prioritization of genes involved in the «endothelial cell apoptotic process» (GO: 0072577) using a training set that included genes associated with lymphedema. The prioritization process was based on the use of well-known methods (ToppGene and Endeavour), as well as on our original approaches, allowing to consider the structure of the gene network of lymphedema, over-represented biological processes and associations of genes with diseases comorbid to lymphedema. It appeared that two genes *GATA2* and *KDR* were simultaneously presented in the list of genes from the «endothelial cell apoptotic process», as well as in the list of genes associated with lymphedema [43, 44]. During the prioritization, these two genes were excluded from the training set and were used as control genes. Thus, the prioritization was performed for 64 genes from the testing set and 67 genes from the training set, associated with lymphedema.

According to the criterion 6 (Additional file 5: Table S5), calculated as an average value of criteria 1–5, it turned out that the *KDR* control gene was on the first place, while the *GATA2* gene was on twelfth. Hypergeometric distribution showed that the enrichment of the top 12 genes by these two control genes is statistically significant (*p*-value = 0.03). It should be noted that a similar analysis, carried out by individual criteria 1–5, showed the absence of any statistically significant enrichment. Thus, the criterion 6, which takes into account five previous criteria, can be considered as the best approach to the genes prioritization.

In the Table 1 are shown top 10 of the highest priority genes according to the criterion 6. Among these top 10 genes SNPs associated with lymphedema are known only for KDR gene [44], which had the first place in the table. For the other genes, to our knowledge, there was no information about any SNPs associated with this disease and these genes can be considered as new candidates for lymphedema susceptibility.

**Table 1:** Top 10 of the highest priority genes according to the criterion 6 calculated as the average value of five criteria

Gene Symbol	Protein name	Gene Id	1	2	3	4 A	4 B	5	6
			ToppGene	Endeavour	GO BP	Gene CTC	Protein CTC	Comorbid	Average
*KDR* ^a^	Vascular Endothelial Growth Factor Receptor 2	3791	1	1	2	1	4	11	1
*TNF*	Tumor Necrosis Factor alpha	7124	20	7	32	2	1	1	2
*TEK*	TEK Receptor Tyrosine Kinase	7010	2	5	4	19,5	11,5	27,5	3
*BMPR2*	Bone Morphogenetic Protein Receptor Type 2	659	15	12	25	9	10	18,5	4
*SERPINE1*	Serpin Family E Member 1	5054	10	27	36,5	9	11,5	3	5
*IL10*	Interleukin 10	3586	19	26	44	5,5	3	3	6
*CD40LG*	CD40 Ligand	959	29	31	20	7	5	11	7
*CCL2*	C-C Motif Chemokine Ligand 2	6347	6	43	46	5,5	6	3	8
*FASLG*	Fas Ligand	356	7	9	40	19,5	27,5	11	9
*ABL1*	ABL Proto-Oncogene 1, Non-Receptor Tyrosine Kinase	25	4	4	42	9	7,5	49	10

^a^Control gene with known associations with lymphedema

Also, it was interesting to consider each remaining criterion separately, even though none of them showed any statistical significance during the evaluation of the enrichment of the top of genes, ranked by these criteria, by the control genes. Thus, both methods of prioritization (ToppGene and Endeavour) determined the *KRD* control gene in the first place in the resulting list. The top 5 of the most priority genes according to both ToppGene (Additional file 6: Table S6) and Endeavour (Additional file 7: Table S7) were *KDR, ABL1* and *TEK*, presented in Table 1. Using the criterion 3 the top 10 included the control gene *KDR* and the *TEK* gene (Additional file 8: Table S8). At the same time, according to criterion 4A (Additional file 9: Table S9), the top 10 included eight genes (*KDR, TNF, IL10, CCL2, CD40LG, ABL1, BMPR2* and *SERPINE1*) from the Table 1. While for the criterion 4B (Additional file 10: Table S10) there were seven such genes in a list (*TNF, IL-10, KDR, CD40LG, CCL2, ABL1* and *BMPR2*). According to the criterion 5, which considers the comorbidity of diseases, the *TNF* gene, associated with six comorbid diseases, was in the first place (Additional file 11: Table S11), the second priority was given to *CCL2, IL10* and *SERPINE1* genes, associated with five comorbid diseases. These genes also appeared to be in the top 10 list (Table 1).

### Verification of the prioritization results using the full-text articles

Jenssen et al., 2001 proposed an approach for automated identification of potential interactions between biological objects, based on an assessment of the statistical significance of the co-occurrence of terms in scientific publications [45]. Because this approach is not implemented in ANDSystem the estimation of the co-occurrence between the analyzed genes and lymphedema can serve as an indirect evidence of the correctness of our prioritization results. It can be expected that the most priority genes would have the highest frequency of mentioning in articles together with lymphedema. As the ANDSystem knowledge base was created by automated analysis of PubMed, to obtain results that could be even more free of the ANDSystem data, we performed a manual analysis of the full-text articles from PubMed Central (Additional file 12: Table S12). It was found that of the 64 of analyzed genes, the 21 genes were statistically significantly more often mentioned in publications together with the “lymphedema” word (*p*-value< 0,05 with FDR correction). 13 out of these 21 genes appeared to be presented among the top 20 genes, ranked according to criterion 6 (Table 2), while the most significant association with lymphedema was observed for the *GATA2* and *KDR* control genes (*p*-value = 10^− 195^ and p-value = 10^− 136^, respectively).

**Table 2:** Characterization of the results of genes prioritization based on the analysis of the frequency of their co-occurrence with lymphedema in the full-text articles

Criterion	Criterion name	Spearman’s Rho correlation	p-value for correlation	Number of control genes in top 20	p-value for enrichment of top 20 genes
1	ToppGene	0,395	0,0012	9	0,133
2	Endeavour	0,437	0,0003	11	0,013
3	GO PB	0,249	0,0464	10	0,047
4A	CTC for genes	0,226	0,0724	11	0,013
4B	CTC for proteins	0,333	0,0072	12	0,003
5	Comorbidity	0,35	0,0046	13	0,0004
6	Average rank	0,529	10^−4^	13	0,0004

According to the hypergeometric distribution, the statistical significance of such enrichment of the top 20 genes had a p-value< 0.0004. It should be noted that only criterion 5, which considers the comorbidity, also had such a low p-value, while the *p*-values of other criteria were higher. At the same time, the highest Spearman’s rank correlation coefficient between the genes ranks and the significance of co-occurrence of the analyzed genes names and lymphedema in the full-text articles (r = 0,529), was observed for the criterion 6 (Table 2).

### Agreement between the prioritization results and gene expression data from the mouse lymphedema model

Lymphedema is characterized by a chronic stasis of lymph in the tissues. In Tabibiazar et al., 2006, the authors performed gene expression profiling by an array in the experimental model of acute postoperative lymphedema associated with lymphatic stagnation in the tails of SKH-1 mice [39]. The following conditions were examined: (1) lymphedema tail skin caused by surgical lymphatic vessel blockage (test); (2) normal tail skin with no intervention (control 1); (3) surgical sham control tail skin - surgical incision with no lymphatic vessel blockage (control 2). Using the data from Tabibiazar et al., 2006 [39] (GEO series GSE4333) 10201 mouse microarray probes differentially expressed between test and control 1, and 2219 probes for test versus control 2 with *p*-value< 0.05 (Additional file 13: Table S13) were detected by GEO2R (http://www.ncbi.nlm.nih.gov/geo/geo2r/). After the mapping of these differentially expressed probes on orthologues human genes from the 10,201 probes 3289 differentially expressed human genes were remained, while of the 2219 probes remained 735 genes. The remaining probes were removed from the analysis due to the lack of any intersections with human genes. After combining these two sets 3494 differentially expressed genes were obtained. It appeared that 13 of 64 analyzed genes were in the combined set.

According to criterion 6, among the top 20 of the highest priority genes there were 8 of 13 differentially expressed genes (*KDR, PLCG1, SERPINE1, CD40LG, IL10, CCL2, PDPK1* and *THBS1*). It was shown by hypergeometric distribution that such enrichment is statistically significant (*p*-value = 0.012, Table 3). However, the greatest enrichment value was observed for criterion 4B. This criterion is based on an assessment of the CTC centrality of the nodes of the gene network, corresponding to proteins.

**Table 3:** Characterization of the prioritization results, obtained using different criteria, based on the analysis of differentially expressed (DE) genes in the murine model of lymphedema

Criterion	Criterion name	Number of DE genes from top 20	p-value of enrichment by DE genes
1	ToppGene	8	0,012
2	Endeavour	4	0,638
3	GO BP	5	0,376
4 A	CTC for genes	9	0,002
4 B	CTC for proteins	10	0,0002
5	Comorbidity	8	0,012
6	Average rank	8	0,012

### Interactions of the top 10 candidates with participants of the lymphedema gene network

**Fig. 3:**
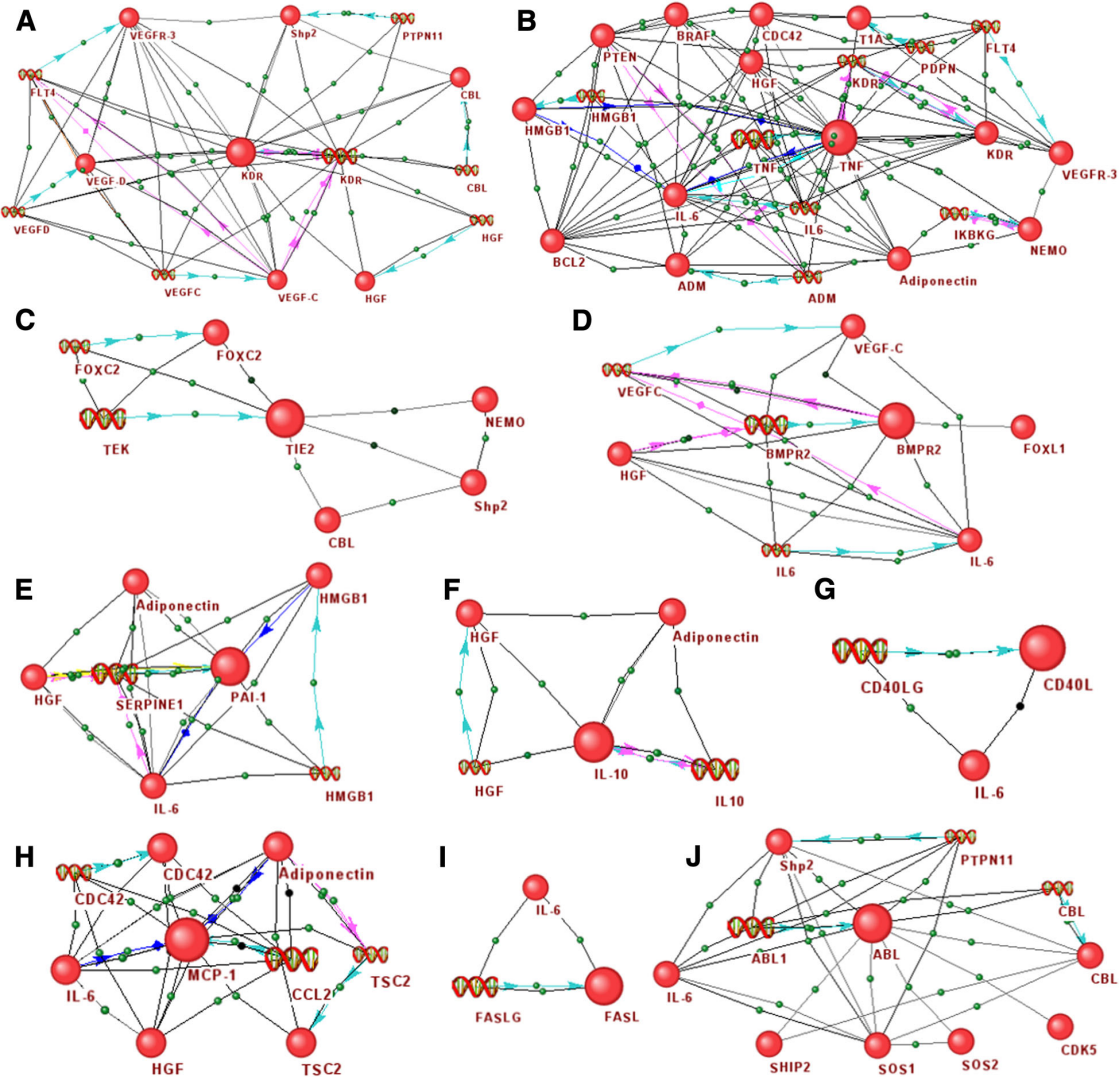
Gene networks describing the interactions of the top 10 of the most promising candidate genes **a** *KDR*, **b** *TNF*, **c** *TEK*, **d** *BMPR2*, **e** *SERPINE1*, **f** *IL10*, **g** *CD40LG*, **h** *CCL2*, **i** *FASLG* and **j** *ABL1* with genes/proteins associated with lymphedema, reconstructed by ANDSystem. Candidate genes and their products are illustrated with large icons

The associative gene networks reconstructed by ANDSystem were used for the analysis of interactions of the top 10 candidate genes, obtained by criterion 6, with the genes/proteins associated with lymphedema (Additional file 14: Table S14, Fig. 3).

The highest priority, according to Table 1, had the control gene *KDR*, which encodes Vascular Endothelial Growth Factor Receptor 2. KDR is one of the main regulators of endothelial cell growth [46], it provides endothelial survival [47], proliferation, migration [48], sprouting [49] and tubular morphogenesis [50, 51]. It is known that a number of polymorphisms in this gene are associated with lymphedema with extreme risk estimates OR > 2, p-value< 0.05 [44]. In the mouse tail lymphedema model, using the quantitative real-time polymerase chain reaction, it was shown that the messenger RNA (mRNA) expression of *KDR* was increased in the group treated by the low-level laser therapy compared to the control [52]. The associative gene network describing the interactions of the *KDR* gene consisted of 14 nodes (Fig. 3a). In particular, the KDR protein had protein-protein interactions with VEGF-C, VEGF-D, VEGFR-3 and CBL [53–56]. It is known that mutations in *VEGFC, VEGFR-3* and *CBL* genes are associated with lymphedema [4, 57–63]. Serum level of VEGF-D was shown to be significantly higher in the group of patients with primary lymphedema compared with controls [64]. It is also known from the literature that the *KDR* and *SHP2* genes have a positive correlation of their expression levels [65]. Mutations in the *SHP2* gene are associated with lymphedema [66]. HGF in combination with VEGF-A can activate VEGFR-2 [67]. Mutations in the *HGF* gene are also associated with lymphedema [68, 69], and increased expression of *HGF* improves lymphedema [70, 71].

The second place in Table 1 belonged to the Tumor Necrosis Factor alpha (*TNF*) gene, which encodes cytokine involved in systemic inflammation [72]. Figure 3B shows the 20 nodes associated with this gene, in particular, TNF was associated with IL-6. It is known from the literature that this protein is capable to activate the expression of the *IL-6* gene [73], increase stability and secretion of IL-6 [74, 75]. In turn, IL-6 has been shown to be increased in models of lymphedema [76]. Another example is the HMGB1 protein, which was also connected to TNF in the associative gene network. It is known that HMGB1 can induce expression of *TNF* [77]. Also studies show the 2.4-fold increased level of HMGB1 in secondary lymphedema [78]. Besides, TNF was found to be associated with adiponectin in the network, a level of which can be reduced in response to the TNF [79]. Shimizu et al., 2013 showed that adiponectin can promote a lymphatic vessel formation resulting in amelioration of lymphedema [80].

The potential role of *TNF* in the molecular mechanisms of lymphedema is discussed in the literature. It is known that TNF can induce endothelial cells apoptosis [81–83]. Földi et al., 2000 showed a decrease in the level of expression of *TNF* after the complex decongestive physiotherapy in patients with peripheral leg lymphedema [84]. Ji, 2007, reports the importance of the role of TNF in the pathophysiological changes of the lymphatic endothelial cells (LECs) and inflammatory lymphangiogenesis [85]. Anuradha et al., 2012, shows the association of inflammatory cytokines (*IL-1β*, *IL-12*, and *TNF*) with pathogenesis of lymphatic filarial infection, which can lead to lymphedema [86]. Jeong et al., 2013 by using the western blot analysis, showed that hyaluronidase treatment of lymphedema in a mouse tail model declined expression of *TNF* [87].

The third place in Table 1 had the *TEK* gene – a receptor tyrosine kinase, involved in a signaling pathway related to the embryonic vascular development [88]. It is known that TEK can reduce proliferation of endothelial cell and apoptosis [89], while mutations in this gene can cause autosomal dominantly inherited forms of venous malformations [90, 91]. The reconstructed gene network contained four interactions of the *TEK* gene with genes/proteins (FOXC2, NEMO, Shp2 and CBL) associated with lymphedema (Fig. 3c). From the literature it is known that the loss of FOXC2 can result in *TEK* downregulation [92] meanwhile, a complete loss or a significant gain of *FOXC2* function can lead to perturbation of lymphatic vessel formation and lymphedema [93]. According to information from the Innatedb [94], HPRD [95] and BioGrid [96] databases, the TEK protein can interact with the NEMO, Shp2 and CBL proteins, mutations in which are associated with lymphedema [63, 66, 97].

The bone morphogenetic protein receptor type II (*BMPR2*) gene – a serine/threonine receptor kinase involved in cell growth and differentiation, osteogenesis and adipogenesis [98] was on the fourth place. A reduced *BMPR2* expression can induce mitochondrial dysfunction of endothelial cells, promoting a pro-inflammatory and pro-apoptotic state [99, 100]. Kim, Kim, 2014 showed that knockdown of *Bmpr2a* and *Bmpr2b* result in lymphatic defects in developing zebrafish [101]. In the constructed associative gene network, *BMPR2* has four interactions with the VEGFC, IL6, HGF and FOXL1 genes/proteins (Fig. 3d). It is known that silencing of *BMPR2* results in down-regulation of *VEGFC* expression [102]. Also, VEGFC plays an important role in the functioning of lymphatic vessels while mutations in this gene are associated with lymphedema [57–60]. Soon et al., 2015 showed that mutations in *BMPR2* lead to higher levels of IL6 [103], which level is increased in lymphedema models [76]. The expression of *BMPR2* was up-regulated by HGF [104]. The increase of expression of *HGF* improves lymphedema [71, 105], and mutations in this gene are associated with the disease [68, 69]. According to the HPRD database, the FOXL1 protein interacts with BMPR2, and mutations in the *FOXL1* gene are associated with lymphedema [106].

Serpin Family E Member 1 (*SERPINE1*), which is the inhibitor of tissue plasminogen activator and urokinase [107] that regulates fibrinolysis [108], appeared to be in the fifth place of the table. The *SERPINE1* gene mediates the anti-apoptotic effect in the endothelial cell [109]. A number of studies have shown the association of *SERPINE1* with the metastasis of tumors in the lymph nodes [110–113]. In the associative gene network, SERPINE1 interacts with four genes/proteins: IL6, HGF, HMGB1 and APN (Fig. 3e). It is known that *SERPINE1* expression decreases with the addition of IL-6 [114] that is increased in lymphedema models [76]. HGF increases *SERPINE1* expression [115], and increase of *HGF* expression improves lymphedema [70, 71]. It was shown that levels of HMGB1 and SERPINE1 had positive correlation [116], while correlation of APN and SERPINE1 was negative [117]. The administration of APN improved the edema of injured tails in the mouse model of lymphedema [80].

The sixth in the Table 1 was an anti-inflammatory cytokine *IL10*, playing an important role in the immunoregulation and inflammation [118]. It was shown that IL-10 significantly blocked endothelial apoptosis [119]. However, other study showed that IL-10 had the capacity to induce macrophage apoptosis [120]. An increased gene expression of *IL10* was found in keratinocytes derived from limb affected by lymphedema [121] and in wounded lymphedematous skin [122]. In the associative network *IL-10* appeared to be connected with the *APN* and *HGF* genes (Fig. 3f). It is known that APN can promote amelioration of lymphedema [80] and is able to significantly increase *IL-10* gene expression and protein secretion [123]. HGF can increase plasma IL-10 concentration [124, 125].

The seventh line in the Table 1 was taken by the *CD40LG* gene, which is expressed in T cells and its product is presented on their surface [126]. CD40LG can bind CD40 on the B cell surface and is involved in T cell proliferation and cytokine production [127]. It is known from the literature that the product of the *CD40LG* gene can increased apoptosis of endothelial cells [128]. This gene was found to be interacting only with IL-6 in the associative gene network (Fig. 3g). Sommer et al., 2009 showed that CD40LG can up-regulate expression of *IL-6* [129].

On the eighth line was the *CCL2* (C-C Motif Chemokine Ligand 2) gene, which encodes the cytokine possessing chemotactic activity for monocytes [130] and basophils [131]. Down-regulation of *CCL2* by miR-495 resulted in inhibited apoptosis of human umbilical vein endothelial cells [132]. In the associative gene network this gene was interacting with 5 genes/proteins (Fig. 2h). Among the genes involved in interactions with *CCL2* were *APN, CDC42, HGF, IL6* and *TSC2*, which are associated with lymphedema [68–71, 76, 80, 133–135]. It is known that APN elevates mRNA and protein level of the CCL2 and stimulates release of CCL2 in primary human monocytes [136]. Ablation of CDC42 induced an overexpression of *CCL2* [137]. Müller et al., 2012 showed that CCL2 can induce HGF [138]. It was shown that IL6 induced CCL2 [139] and the IL6 level positively correlated with the CCL2 [140]. Loss of *TSC2* function can result in overexpression of *CCL2* [141].

The *FASLG* gene, located on the ninth row of the Table 1, is a member of tumor necrosis factor superfamily [142, 143]. *FASLG* is encoding transmembrane protein Fas ligand that is responsible for the induction of apoptosis triggered by binding to FAS [144–146]. Abnormal *FASLG* expression is associated with a metastasis of tumors in the lymph nodes [147–149]. In the reconstructed associative gene network *FASLG* appeared to be associated only with IL6 (Fig. 2i). It is known that FASLG stimulation can enhanced IL-6 release [150].

On the tenth line of the Table 1 was the *ABL1* protooncogene, which is encoding a tyrosine kinase protein, participating in cell division and differentiation, adhesion, and response to oxidative stress [151]. *ABL1* is involved in mediating endothelial apoptosis [152]. In reconstructed gene network *ABL1* was associated with 7 genes/proteins (Fig. 3j): CBL, CDK5, IL6, SHIP2, Shp2, SOS1 and SOS2. It is known that mutations in these genes are associated with lymphedema [63, 68, 153–157]. The Innatedb, HPRD and BioGrid databases contain data describing protein-protein interactions of ABL1 with CBL, CDK5, SHIP2, Shp2, SOS1 and SOS2. Also, ABL1 positively controls *IL-6* expression [158].

## Conclusion

In this work, we performed a search for new potential participants of lymphedema molecular-genetic mechanisms based on the prioritization of genes involved in endothelial cell apoptosis. Six criteria, including the well-known ToppGene and Endeavour methods, as well as our original approaches [19, 20] were used. The use of original methods allowed taking into account the overrepresented Gene Ontology biological processes, structural features of the associative gene network of lymphedema and endothelial apoptosis, as well as diseases comorbid to lymphedema. Verification of the prioritization quality using three different criteria showed significant enrichment of the most priority genes with known genes, which have simultaneous interactions with lymphedema and endothelial apoptosis, as well as with genes differentially expressed in the murine model of lymphedema. Among genes, involved in endothelial cell apoptosis, *TNF, TEK, BMPR2, SERPINE1, IL10, CD40LG, CCL2, FASLG* and *ABL1* were identified as the most promising candidates that can be used for planning the experiments concerning their possible associations with lymphedema. Besides, analysis of the function of these genes can help in understanding the molecular-genetic role of endothelial cell apoptosis in lymphedema.

## Additional files

Additional file 1: **Table S1.** Lists of genes associated with lymphedema. (XLSX 12 kb)

Additional file 2: **Table S2.** Results of gene ontology enrichment analysis. (XLSX 14 kb)

Additional file 3: **Table S3.** Lists of diseases comorbid to lymphedema. (XLSX 11 kb)

Additional file 4: **Table S4.** Values of betweenness centrality of nodes of lymphedema associative gene network. (XLSX 16 kb)

Additional file 5: **Table S5.** Ranks of analyzed genes. (XLSX 23 kb)

Additional file 6: **Table S6.** Results of prioritization according to criterion 1. (XLSX 30 kb)

Additional file 7: **Table S7.** Results of prioritization according to criterion 2. (XLSX 33 kb)

Additional file 8: **Table S8.** Results of prioritization according to criterion 3. (XLSX 17 kb)

Additional file 9: **Table S9.** Results of prioritization according to criterion 4A. (XLSX 15 kb)

Additional file 10: **Table S10.** Results of prioritization according to criterion 4B. (XLSX 16 kb)

Additional file 11: **Table S11.** Results of prioritization according to criterion 5. (XLSX 17 kb)

Additional file 12: **Table S12.** Frequency of mentioning analyzed gene names together with lymphedema in in full-text articles. (XLSX 13 kb)

Additional file 13: **Table S13.** Differentially expressed genes in mouse model of lymphedema. (XLSX 471 kb)

Additional file 14: **Table S14.** Interactions of top 10 most promising candidate genes with genes/proteins associated with lymphedema. (XLSX 173 kb)

## Abbreviations

CTC: Cross-talk centrality
DE: Differentially expressed
FDR: False discovery rate
GO PB: Gene Ontology biological processes
GO: Gene Ontology
LECs: Lymphatic endothelial cells
mRNA: Messenger RNA

## References

[1] Farinola N, Piller NB (2007). CYP2A6 polymorphisms: is there a role for pharmacogenomics in preventing coumarin-induced hepatotoxicity in lymphedema patients?. Pharmacogenomics.

[2] Poveshchenko AF, Nimaev VV, Lubarsky MS, Konenkov VI (2010). Medical and genetical aspects of lymphedema. Med Genet.

[3] Szuba A, Rockson SG (1998). Lymphedema: classification, diagnosis and therapy. Vasc Med.

[4] Mendola A, Schlögel MJ, Ghalamkarpour A, Irrthum A, Nguyen HL, Fastré E (2013). Mutations in the VEGFR3 signaling pathway explain 36% of familial lymphedema. Mol Syndromol.

[5] Brouillard P, Boon L, Vikkula M (2014). Genetics of lymphatic anomalies. J Clin Invest.

[6] Gupta A, Moore JA (2018). Lymphedema. JAMA Oncol.

[7] Leung G, Baggott C, West C, Elboim C, Paul SM, Cooper BA (2014). Cytokine candidate genes predict the development of secondary lymphedema following breast cancer surgery. Lymphat Res Biol.

[8] Miaskowski C, Dodd M, Paul SM, West C, Hamolsky D, Abrams G (2013). Lymphatic and angiogenic candidate genes predict the development of secondary lymphedema following breast cancer surgery. PLoS One.

[9] Shaitelman SF, Cromwell KD, Rasmussen JC, Stout NL, Armer JM, Lasinski BB (2015). Recent progress in cancer-related lymphedema treatment and prevention. CA Cancer J Clin.

[10] Olszewski WL (2003). Pathophysiological aspects of lymphedema of human limbs: I. Lymph protein composition. Lymphat Res Biol.

[11] Tian W, Rockson SG, Jiang X, Kim J, Begaye A, Shuffle EM, Tu AB, Cribb M, Nepiyushchikh Z, Feroze AH, Zamanian RT (2017). Leukotriene B4 antagonism ameliorates experimental lymphedema. Sci Transl Med.

[12] Kerr J, Wyllie A, Currie A (1972). Apoptosis: a basic biological phenomenon with wide-ranging implications in tissue kinetics. Br J Cancer.

[13] Wang JF, Zhang X, Groopman JE (2004). Activation of vascular endothelial growth factor receptor-3 and its downstream signaling promote cell survival under oxidative stress. J Biol Chem.

[14] Ogunbiyi S, Chinien G, Field D, Humphries J, Burand K, Sawyer B (2011). Smith, for the London lymphedema consortium a. Molecular characterization of dermal lymphatic endothelial cells from primary lymphedema skin. Lymphat Res Biol.

[15] Chen J, Bardes EE, Aronow BJ, Jegga AG (2009). ToppGene Suite for gene list enrichment analysis and candidate gene prioritization. Nucleic Acids Res.

[16] Moreau Y, Tranchevent LC (2012). Computational tools for prioritizing candidate genes: boosting disease gene discovery. Nat Rev Genet.

[17] Guney E, Oliva B (2012). Exploiting protein-protein interaction networks for genome-wide disease-gene prioritization. PLoS One.

[18] Tranchevent LC, Ardeshirdavani A, ElShal S, Alcaide D, Aerts J, Auboeuf D (2016). Candidate gene prioritization with Endeavour. Nucleic Acids Res.

[19] Saik OV, Demenkov PS, Ivanisenko TV, Bragina EY, Freidin MB, Goncharova IA (2018). Novel candidate genes important for asthma and hypertension comorbidity revealed from associative gene networks. BMC Med Genet.

[20] Yankina MA, Saik OV, Demenkov PS, Khusnutdinova EK, Rogaev EI, Lavrik IN (2018). Analysis of the interactions of neuronal apoptosis genes in the associative gene network of Parkinson's disease. Vavilovskii Zhurnal Genetiki I SelektsII.

[21] Demenkov PS, Ivanisenko TV, Kolchanov NA, Ivanisenko VA (2012). ANDVisio: a new tool for graphic visualization and analysis of literature mined associative gene networks in the ANDSystem. In Silico Biol.

[22] Ivanisenko VA, Saik OV, Ivanisenko NV, Tiys ES, Ivanisenko TV, Demenkov PS (2015). ANDSystem: an associative network discovery system for automated literature mining in the field of biology. BMC Syst Biol.

[23] Glotov AS, Tiys ES, Vashukova ES, Pakin VS, Demenkov PS, Saik OV (2015). Molecular association of pathogenetic contributors to pre-eclampsia (pre-eclampsia associome). BMC Syst Biol.

[24] Bragina EY, Tiys ES, Freidin MB, Koneva LA, Demenkov PS, Ivanisenko VA (2014). Insights into pathophysiology of dystropy through the analysis of gene networks: an example of bronchial asthma and tuberculosis. Immunogenetics.

[25] Mattingly CJ, Rosenstein MC, Colby GT, Forrest JN, Boyer JL (2006). The comparative Toxicogenomics database (CTD): a resource for comparative toxicological studies. J Exp Zool A Comp Exp Biol.

[26] Rappaport N, Twik M, Plaschkes I, Nudel R, Iny Stein T, Levitt J (2016). MalaCards: an amalgamated human disease compendium with diverse clinical and genetic annotation and structured search. Nucleic Acids Res.

[27] Kanehisa M, Furumichi M, Tanabe M, Sato Y, Morishima K (2016). KEGG: new perspectives on genomes, pathways, diseases and drugs. Nucleic Acids Res.

[28] Köhler S, Vasilevsky NA, Engelstad M, Foster E, McMurry J, Aymé S (2016). The human phenotype ontology in 2017. Nucleic Acids Res.

[29] Piñero J, Bravo À, Queralt-Rosinach N, Gutiérrez-Sacristán A, Deu-Pons J, Centeno E, García-García J, Sanz F, Furlong LI. DisGeNET: a comprehensive platform integrating information on human disease-associated genes and variants. Nucleic Acids Res. 2016;45(D1):D833-D839. 10.1093/nar/gkw943 PMC5210640 27924018

[30] Usmonov DB, Saik OV, Nimaev VV. The possibilities of bioinformatic analysis in the study of the pathogenesis of lymphatic dysplasia: Engineering, Computer and Information Sciences (SIBIRCON). 2017 International multi-conference on 2017 Sep 18. Hoboken: Wiley-IEEE Press; 2017. p. 512–4.

[31] Huang DW, Sherman BT, Lempicki RA (2008). Systematic and integrative analysis of large gene lists using DAVID bioinformatics resources. Nat Protoc.

[32] Hagberg A, Swart P, S Chult D (2008). Exploring network structure, dynamics, and function using NetworkX.

[33] Oliphant TE (2007). Python for scientific computing. Comput Sci Eng.

[34] Carbon S, Ireland A, Mungall CJ, Shu S, Marshall B, Lewis S (2008). AmiGO hub, web presence working group. AmiGO: online access to ontology and annotation data. Bioinformatics.

[35] Feinstein AR (1970). The pre-therapeutic classification of co-morbidity in chronic disease. J Chronic Dis.

[36] Puzyrev VP (2015). Genetic bases of human comorbidity. Russ J Genet.

[37] Spearman C (1906). ‘Footrule’for measuring correlation. Br J Psychol 1904–1920.

[38] Radua J, Mataix-Cols D (2012). Meta-analytic methods for neuroimaging data explained. Biology of mood & anxiety disorders.

[39] Tabibiazar R, Cheung L, Han J, Swanson J, Beilhack A, An A (2006). Inflammatory manifestations of experimental lymphatic insufficiency. PLoS Med.

[40] Saito Y, Nakagami H, Kaneda Y, Morishita R (2013). Lymphedema and therapeutic lymphangiogenesis. Biomed Res Int.

[41] Yoon YS, Murayama T, Gravereaux E, Tkebuchava T, Silver M, Curry C (2003). VEGF-C gene therapy augments postnatal lymphangiogenesis and ameliorates secondary lymphedema. J Clin Invest.

[42] Coso S, Zeng Y, Sooraj D, Williams ED (2011). Conserved signaling through vascular endothelial growth (VEGF) receptor family members in murine lymphatic endothelial cells. Exp Cell Res.

[43] Kazenwadel J, Secker GA, Liu YJ, Rosenfeld JA, Wildin RS, Cuellar-Rodriguez J (2011). Loss-of-function germline GATA2 mutations in patients with MDS/AML or MonoMAC syndrome and primary lymphedema reveal a key role for GATA2 in the lymphatic vasculature. Blood.

[44] Newman B, Lose F, Kedda MA, Francois M, Ferguson K, Janda M (2012). Possible genetic predisposition to lymphedema after breast cancer. Lymphat Res Biol.

[45] Jenssen TK, Lægreid A, Komorowski J, Hovig E (2001). A literature network of human genes for high-throughput analysis of gene expression. Nat Genet.

[46] Liu Z, Qi L, Li Y, Zhao X, Sun B (2017). VEGFR2 regulates endothelial differentiation of colon cancer cells. BMC Cancer.

[47] Ou JM, Yu ZY, Qiu MK, Dai YX, Dong Q, Shen J (2014). Knockdown of VEGFR2 inhibits proliferation and induces apoptosis in hemangioma-derived endothelial cells. Eur J Histochem.

[48] Liu Y, Qiao Y, Hu C, Liu L, Zhou L, Liu B (2016). VEGFR2 inhibition by RNA interference affects cell proliferation, migration, invasion, and response to radiation in Calu-1 cells. Clin Transl Oncol.

[49] Gaengel K, Niaudet C, Hagikura K, Laviña B, Muhl L, Hofmann JJ (2012). The sphingosine-1-phosphate receptor S1PR1 restricts sprouting angiogenesis by regulating the interplay between VE-cadherin and VEGFR2. Dev Cell.

[50] van Tuyl M, Groenman F, Wang J, Kuliszewski M, Liu J, Tibboel D (2007). Angiogenic factors stimulate tubular branching morphogenesis of sonic hedgehog-deficient lungs. Dev Biol.

[51] Mellberg S, Dimberg A, Bahram F, Hayashi M, Rennel E, Ameur A (2009). Transcriptional profiling reveals a critical role for tyrosine phosphatase VE-PTP in regulation of VEGFR2 activity and endothelial cell morphogenesis. FASEB J.

[52] Jang DH, Song DH, Chang EJ, Jeon JY (2016). Anti-inflammatory and lymphangiogenetic effects of low-level laser therapy on lymphedema in an experimental mouse tail model. Lasers Med Sci.

[53] Enholm B, Karpanen T, Jeltsch M, Kubo H, Stenbacz F, Prevo R (2001). Adenoviral expression of vascular endothelial growth factor-C induces lymphangiogenesis in the skin. Circ Res.

[54] Partanen TA, Arola J, Saaristo A, Jussila L, Ora A, Miettinen M (2000). VEGF-C and VEGF-D expression in neuroendocrine cells and their receptor, VEGFR-3, in fenestrated blood vessels in human tissues. FASEB J.

[55] Achen MG, Roufail S, Domagala T, Catimel B, Nice EC, Geleick DM (2000). Monoclonal antibodies to vascular endothelial growth factor-D block its interactions with both VEGF receptor-2 and VEGF receptor-3. Eur J Biochem.

[56] Meyer RD, Sacks DB, Rahimi N (2008). IQGAP1-dependent signaling pathway regulates endothelial cell proliferation and angiogenesis. PLoS One.

[57] Shin M, Male I, Beane TJ, Villefranc JA, Kok FO, Zhu LJ, Lawson ND. Vegfc acts through ERK to induce sprouting and differentiation of trunk lymphatic progenitors. Development. 2016;143(20):3785-3795. 10.1242/dev.137901 PMC5087638 27621059

[58] Le Guen L, Karpanen T, Schulte D, Harris NC, Koltowska K, Roukens G, Bower NI, Van Impel A, Stacker SA, Achen MG, Schulte-Merker S. Ccbe1 regulates Vegfc-mediated induction of Vegfr3 signaling during embryonic lymphangiogenesis. Development. 2014;141(6):1239-49. 10.1242/dev.100495 24523457

[59] Gousopoulos E, Proulx ST, Bachmann SB, Dieterich LC, Scholl J, Karaman S (2017). An important role of VEGF-C in promoting lymphedema development. J Investig Dermatol.

[60] Fastré E, Lanteigne LE, Helaers R, Giacalone G, Revencu N, Dionyssiou D (2018). Splice-site mutations in VEGFC cause loss of function and nonne-Milroy-like primary lymphedema. Clin Genet.

[61] Saaristo A, Veikkola T, Tammela T, Enholm B, Karkkainen MJ, Pajusola K (2002). Lymphangiogenic gene therapy with minimal blood vascular side effects. J Exp Med.

[62] Mizuno S, Yamada Y, Yamada K, Nomura N, Wakamatsu N (2005). Clinical variability in a Japanese hereditary lymphedema type I family with an FLT4 mutation. Congenit Anom.

[63] Hanson HL, Wilson MJ, Short JP, Chioza BA, Crosby AH, Nash RM (2014). Germline CBL mutation associated with a Noonan-like syndrome with primary lymphedema and teratoma associated with acquired uniparental isodisomy of chromosome 11q23. Am J Med Genet A.

[64] Fink AM, Kaltenegger I, Schneider B, Fruhauf J, Jurecka W, Steiner A (2004). Serum level of VEGF-D in patients with primary lymphedema. Lymphology.

[65] Tang C, Luo D, Yang H, Wang Q, Zhang R, Liu G (2013). Expression of SHP2 and related markers in non–small cell lung cancer: a tissue microarray study of 80 cases. Appl Immunohistochem Mol Morphol.

[66] Yeang CH, Ma GC, Shih JC, Yang YS, Chen CP, Chang SP (2012). Genome-wide gene expression analysis implicates the immune response and lymphangiogenesis in the pathogenesis of fetal chylothorax. PLoS One.

[67] Sulpice E, Ding S, Muscatelli-Groux B, Bergé M, Han ZC, Plouet J (2009). Cross-talk between the VEGF-A and HGF signalling pathways in endothelial cells. Biol Cell.

[68] Finegold DN, Schacht V, Kimak MA, Lawrence EC, Foeldi E, Karlsson JM (2008). HGF and MET mutations in primary and secondary lymphedema. Lymphat Res Biol.

[69] Michelini S, Vettori A, Maltese PE, Cardone M, Bruson A, Fiorentino A (2016). Genetic screening in a large cohort of italian patients affected by primary lymphedema using a next generation sequencing (NGS) approach. Lymphology.

[70] Saito Y, Nakagami H, Morishita R, Takami Y, Kikuchi Y, Hayashi H (2006). Transfection of human hepatocyte growth factor gene ameliorates secondary lymphedema via promotion of lymphangiogenesis. Circulation.

[71] Lee CY, Kang JY, Lim S, Ham O, Chang W, Jang DH (2016). Hypoxic conditioned medium from mesenchymal stem cells promotes lymphangiogenesis by regulation of mitochondrial-related proteins. Stem Cell Res Ther.

[72] Popa C, Netea MG, Van Riel PL, Van Der Meer JW, Stalenhoef AF (2007). The role of TNF-α in chronic inflammatory conditions, intermediary metabolism, and cardiovascular risk. J Lipid Res.

[73] Tomita N, Morishita R, Tomita S, Kaneda Y, Higaki J, Ogihara T (2001). Inhibition of TNF-α, induced cytokine and adhesion molecule. Nephron Exp Nephrol.

[74] Shimada M, Andoh A, Hata K, Tasaki K, Araki Y, Fujiyama Y (2002). IL-6 secretion by human pancreatic periacinar myofibroblasts in response to inflammatory mediators. J Immunol.

[75] Lee C, Oh JI, Park J, Choi JH, Bae EK, Lee HJ (2013). TNFα mediated IL-6 secretion is regulated by JAK/STAT pathway but not by MEK phosphorylation and AKT phosphorylation in U266 multiple myeloma cells. Biomed Res Int.

[76] Cuzzone DA, Weitman ES, Albano NJ, Ghanta S, Savetsky IL, Gardenier JC (2014). IL-6 regulates adipose deposition and homeostasis in lymphedema. Am J Phys Heart Circ Phys.

[77] Barkauskaite V, Ek M, Popovic K, Harris HE, Wahren-Herlenius M, Nyberg F (2007). Translocation of the novel cytokine HMGB1 to the cytoplasm and extracellular space coincides with the peak of clinical activity in experimentally UV-induced lesions of cutaneous lupus erythematosus. Lupus.

[78] Zampell JC, Yan A, Avraham T, Andrade V, Malliaris S, Aschen S (2011). Temporal and spatial patterns of endogenous danger signal expression after wound healing and in response to lymphedema. Am J Phys Cell Phys.

[79] Wang B, Trayhurn P (2006). Acute and prolonged effects of TNF-α on the expression and secretion of inflammation-related adipokines by human adipocytes differentiated in culture. Pflugers Arch.

[80] Shimizu Y, Shibata R, Ishii M, Ohashi K, Kambara T, Uemura Y (2013). Adiponectin-mediated modulation of lymphatic vessel formation and lymphedema. J Am Heart Assoc.

[81] Mariño E, Cardier JE (2003). Differential effect of IL-18 on endothelial cell apoptosis mediated by TNF-α and Fas (CD95). Cytokine..

[82] Lejeune FJ, Rüegg C (2006). Recombinant human tumor necrosis factor: an efficient agent for cancer treatment. Bull Cancer.

[83] Acquavella N, Quiroga MF, Wittig O, Cardier JE (2010). Effect of simvastatin on endothelial cell apoptosis mediated by Fas and TNF-α. Cytokine..

[84] Foldi E, Sauerwald A, Hennig B (2000). Effect of complex decongestive physiotherapy on gene expression for the inflammatory response in peripheral lymphedema. Lymphology..

[85] Ji RC (2007). Lymphatic endothelial cells, inflammatory lymphangiogenesis, and prospective players. Curr Med Chem.

[86] Anuradha R, George PJ, Kumar NP, Fay MP, Kumaraswami V, Nutman TB (2012). Circulating microbial products and acute phase proteins as markers of pathogenesis in lymphatic filarial disease. PLoS Pathog.

[87] Jeong HJ, Roh K, Kim G, Kim Y, Lee J, Lee M (2013). Hyaluronidase treatment of acute lymphedema in a mouse tail model. Lymphology..

[88] Dumont DJ, Gradwohl G, Fong GH, Puri MC, Gertsenstein M, Auerbach A (1994). Dominant-negative and targeted null mutations in the endothelial receptor tyrosine kinase, tek, reveal a critical role in vasculogenesis of the embryo. Genes Dev.

[89] Hu HT, Huang YH, Chang YA, Lee CK, Jiang MJ, Wu LW (2008). Tie2-R849W mutant in venous malformations chronically activates a functional STAT1 to modulate gene expression. J Investig Dermatol.

[90] Frigerio A, Stevenson DA, Grimmer JF (2012). The genetics of vascular anomalies. Curr Opin Otolaryngol Head Neck Surg.

[91] Yadav P, De Castro DK, Waner M, Meyer L, Fay A (2013). Vascular anomalies of the head and neck: a review of genetics. Semin Ophthalmol.

[92] Thomson BR, Heinen S, Jeansson M, Ghosh AK, Fatima A, Sung HK (2014). A lymphatic defect causes ocular hypertension and glaucoma in mice. J Clin Invest.

[93] Tavian D, Missaglia S, Maltese PE, Michelini S, Fiorentino A, Ricci M (2016). FOXC2 disease-mutations identified in lymphedema-distichiasis patients cause both loss and gain of protein function. Oncotarget.

[94] Breuer K, Foroushani AK, Laird MR, Chen C, Sribnaia A, Lo R (2012). InnateDB: systems biology of innate immunity and beyond—recent updates and continuing curation. Nucleic Acids Res.

[95] Keshava Prasad TS, Goel R, Kandasamy K, Keerthikumar S, Kumar S, Mathivanan S, Telikicherla D, Raju R, Shafreen B, Venugopal A, Balakrishnan L (2008). Human protein reference database—2009 update. Nucleic Acids Res.

[96] Stark C, Breitkreutz BJ, Reguly T, Boucher L, Breitkreutz A, Tyers M (2006). BioGRID: a general repository for interaction datasets. Nucleic Acids Res.

[97] Roberts CM, Angus JE, Leach IH, McDermott EM, Walker DA, Ravenscroft JC (2010). A novel NEMO gene mutation causing osteopetrosis, lymphoedema, hypohidrotic ectodermal dysplasia and immunodeficiency (OL-HED-ID). Eur J Pediatr.

[98] Xiao YT, Xiang LX, Shao JZ (2007). Bone morphogenetic protein. Biochem Biophys Res Commun.

[99] Spiekerkoetter E, Tian X, Cai J, Hopper RK, Sudheendra D, Li CG (2013). FK506 activates BMPR2, rescues endothelial dysfunction, and reverses pulmonary hypertension. J Clin Invest.

[100] Diebold I, Hennigs JK, Miyagawa K, Li CG, Nickel NP, Kaschwich M (2015). BMPR2 preserves mitochondrial function and DNA during reoxygenation to promote endothelial cell survival and reverse pulmonary hypertension. Cell Metab.

[101] Kim JD, Kim J (2014). Alk3/Alk3b and Smad5 mediate BMP signaling during lymphatic development in zebrafish. Mol Cells.

[102] Zeng P, Cai S, Zhang JN, Yi FM, Jiang WM, Wu JB (2014). Effects of siRNA targeting BMPR-II on the biological activities of human liver cancer cells and its mechanism. Cancer Cell Int.

[103] Soon E, Crosby A, Southwood M, Yang P, Tajsic T, Toshner M (2015). Bone morphogenetic protein receptor type II deficiency and increased inflammatory cytokine production. A gateway to pulmonary arterial hypertension. Am J Respir Crit Care Med.

[104] Ye L, Lewis-Russell JM, Davies G, Sanders AJ, Kynaston H, Jiang WG (2007). Hepatocyte growth factor up-regulates the expression of the bone morphogenetic protein (BMP) receptors, BMPR-IB and BMPR-II, in human prostate cancer cells. Int J Oncol.

[105] Saito Y, Nakagami H, Morishita R (2007). Transfection of human hepatocyte growth factor gene ameliorates secondary lymphedema via promotion of lymphangiogenesis. J Vasc Surg.

[106] Butler MG, Dagenais SL, Garcia-Perez JL, Brouillard P, Vikkula M, Strouse P (2012). Microcephaly, intellectual impairment, bilateral vesicoureteral reflux, distichiasis, and glomuvenous malformations associated with a 16q24. 3 contiguous gene deletion and a Glomulin mutation. Am J Med Genet A.

[107] Ye Y, Vattai A, Zhang X, Zhu J, Thaler CJ, Mahner S (2017). Role of plasminogen activator inhibitor type 1 in pathologies of female reproductive diseases. Int J Mol Sci.

[108] Chen R, Yan J, Liu P, Wang Z, Wang C (2017). Plasminogen activator inhibitor links obesity and thrombotic cerebrovascular diseases: the roles of PAI-1 and obesity on stroke. Metab Brain Dis.

[109] Yao H, He G, Chen C, Yan S, Lu L, Song L (2017). PAI1: a novel PP1-interacting protein that mediates human plasma's anti-apoptotic effect in endothelial cells. J Cell Mol Med.

[110] Eljuga D, Razumovic JJ, Bulic K, Petrovecki M, Draca N, Bulic SO (2011). Prognostic importance of PAI-1 in node negative breast cancer patients—results after 10 years of follow up. Pathol Res Pract.

[111] Dhanda J, Triantafyllou A, Liloglou T, Kalirai H, Lloyd B, Hanlon R (2014). SERPINE1 and SMA expression at the invasive front predict extracapsular spread and survival in oral squamous cell carcinoma. Br J Cancer.

[112] Zhang Y, Zhang YL, Chen HM, Pu HW, Ma WJ, Li XM (2014). Expression of Bmi-1 and PAI-1 in esophageal squamous cell carcinoma. World J Gastroenterol: WJG.

[113] Sang Y, Chen MY, Luo D, Zhang RH, Wang L, Li M (2015). TEL2 suppresses metastasis by down-regulating SERPINE1 in nasopharyngeal carcinoma. Oncotarget.

[114] Umemura K, Ishioka SI, Endo T, Ezaka Y, Takahashi M, Saito T (2013). Roles of microRNA-34a in the pathogenesis of placenta accreta. J Obstet Gynaecol Res.

[115] Imagawa S, Fujii S, Dong J, Furumoto T, Kaneko T, Zaman T (2006). Hepatocyte growth factor regulates E box–dependent plasminogen activator inhibitor type 1 gene expression in HepG2 liver cells. Arterioscler Thromb Vasc Biol.

[116] Nomura S, Maeda Y, Ishii K, Katayama Y, Yagi H, Fujishima N (2016). Relationship between HMGB1 and PAI-1 after allogeneic hematopoietic stem cell transplantation. J Blood Med.

[117] Nomura S, Taniura T, Shouzu A, Omoto S, Inami N, Fujita S (2012). Effects of pitavastatin on plasminogen activator inhibitor-1 in hyperlipidemic patients. Int J General Med.

[118] Hedrich CM, Bream JH (2010). Cell type-specific regulation of IL-10 expression in inflammation and disease. Immunol Res.

[119] Yin Y, Liu W, Ji G, Dai Y (2013). The essential role of p38 MAPK in mediating the interplay of oxLDL and IL-10 in regulating endothelial cell apoptosis. Eur J Cell Biol.

[120] Wang ZQ, Bapat AS, Rayanade RJ, Dagtas AS, Hoffmann MK (2001). Interleukin-10 induces macrophage apoptosis and expression of CD16 (FcγRIII) whose engagement blocks the cell death programme and facilitates differentiation. Immunology.

[121] Baroni A, Buommino E, Piccolo V, Chessa MA, Russo T, Cozza V (2014). Alterations of skin innate immunity in lymphedematous limbs: correlations with opportunistic diseases. Clin Dermatol.

[122] Kimura T, Sugaya M, Blauvelt A, Okochi H, Sato S (2013). Delayed wound healing due to increased interleukin-10 expression in mice with lymphatic dysfunction. J Leukoc Biol.

[123] Kumada M, Kihara S, Ouchi N, Kobayashi H, Okamoto Y, Ohashi K (2004). Adiponectin specifically increased tissue inhibitor of metalloproteinase-1 through interleukin-10 expression in human macrophages. Circulation.

[124] Warzecha Z, Dembiński A, Ceranowicz P, Konturek SJ, Tomaszewska R, Stachura J (2004). Inhibition of cyclooxygenase-2 reduces the protective effect of hepatocyte growth factor in experimental pancreatitis. Eur J Pharmacol.

[125] Chen PM, Liu KJ, Hsu PJ, Wei CF, Bai CH, Ho LJ (2014). Induction of immunomodulatory monocytes by human mesenchymal stem cell-derived hepatocyte growth factor through ERK1/2. J Leukoc Biol.

[126] Mikolajczak SA, Ma BY, Yoshida T, Yoshida R, Kelvin DJ, Ochi A (2004). The modulation of CD40 ligand signaling by transmembrane CD28 splice variant in human T cells. J Exp Med.

[127] Blotta MH, Marshall JD, DeKruyff RH, Umetsu DT (1996). Cross-linking of the CD40 ligand on human CD4+ T lymphocytes generates a costimulatory signal that up-regulates IL-4 synthesis. J Immunol.

[128] Wu CF, Huang FD, Sui RF, Sun JX (2012). Preeclampsia serum upregulates CD40/CD40L expression and induces apoptosis in human umbilical cord endothelial cells. Reprod Biol Endocrinol.

[129] Sommer S, Pudrith CB, Colvin CJ, Coussens PM (2009). Mycobacterium avium subspecies paratuberculosis suppresses expression of IL-12p40 and iNOS genes induced by signalling through CD40 in bovine monocyte-derived macrophages. Vet Immunol Immunopathol.

[130] Ogilvie P, Paoletti S, Clark-Lewis I, Uguccioni M (2003). Eotaxin-3 is a natural antagonist for CCR2 and exerts a repulsive effect on human monocytes. Blood..

[131] Kuna P, Reddigari SR, Rucinski D, Oppenheim JJ, Kaplan AP (1992). Monocyte chemotactic and activating factor is a potent histamine-releasing factor for human basophils. J Exp Med.

[132] Liu D, Zhang XL, Yan CH, Li Y, Tian XX, Zhu N (2015). MicroRNA-495 regulates the proliferation and apoptosis of human umbilical vein endothelial cells by targeting chemokine CCL2. Thromb Res.

[133] Takenouchi T, Okamoto N, Ida S, Uehara T, Kosaki K (2016). Further evidence of a mutation in CDC42 as a cause of a recognizable syndromic form of thrombocytopenia. Am J Med Genet A.

[134] Kacerovska D, Kerl K, Michal M, Filipova H, Vrtel R, Vanecek T (2012). Giant angiofibromas in tuberous sclerosis complex: a possible role for localized lymphedema in their pathogenesis. J Am Acad Dermatol.

[135] Navarre P, Poitras B (2014). Lymphoedema in tuberous sclerosis: case report and review of the literature. J Pediatr Orthop.

[136] Neumeier M, Bauer S, Brühl H, Eisinger K, Kopp A, Abke S (2011). Adiponectin stimulates release of CCL2, −3, −4 and −5 while the surface abundance of CCR2 and −5 is simultaneously reduced in primary human monocytes. Cytokine..

[137] Deroanne CF, Hamelryckx D, Ho TG, Lambert CA, Catroux P, Lapière CM (2005). Cdc42 downregulates MMP-1 expression by inhibiting the ERK1/2 pathway. J Cell Sci.

[138] Müller AM, Jun E, Conlon H, Sadiq SA (2012). Cerebrospinal hepatocyte growth factor levels correlate negatively with disease activity in multiple sclerosis. J Neuroimmunol.

[139] Thomas M, Bayha C, Klein K, Müller S, Weiss TS, Schwab M (2015). The truncated splice variant of peroxisome proliferator-activated receptor alpha, PPARα-tr, autonomously regulates proliferative and pro-inflammatory genes. BMC Cancer.

[140] Cheung CM, Vania M, Ang M, Chee SP, Li J (2012). Comparison of aqueous humor cytokine and chemokine levels in diabetic patients with and without retinopathy. Mol Vis.

[141] Li S, Takeuchi F, Wang JA, Fuller C, Pacheco-Rodriguez G, Moss J (2005). MCP-1 overexpressed in tuberous sclerosis lesions acts as a paracrine factor for tumor development. J Exp Med.

[142] Ruiz-García R, Mora S, Lozano-Sánchez G, Martínez-Lostao L, Paz-Artal E, Ruiz-Contreras J, Anel A, González-Granado LI, Moreno D, Allende LM. Decreased activation-induced cell death by EBV-transformed B cells from a patient with autoimmune lymphoproliferative syndrome caused by a novel FASLG mutation. Pediatr Res. 2015;78(6):603-8. 10.1038/pr.2015.170 26334989

[143] Schneider P, Bodmer JL, Holler N, Mattmann C, Scuderi P, Terskikh A (1997). Characterization of Fas (Apo-1, CD95)-fas ligand interaction. J Biol Chem.

[144] Kokkonen TS, Augustin MT, Mäkinen JM, Kokkonen J, Karttunen TJ (2004). High endothelial venules of the lymph nodes express Fas ligand. J Histochem Cytochem.

[145] Verma RK, Gunda V, Pawar SC, Sudhakar YA (2013). Extra cellular matrix derived metabolite regulates angiogenesis by FasL mediated apoptosis. PLoS One.

[146] Gong Q, Qiu S, Li S, Ma Y, Chen M, Yao Y (2014). Proapoptotic PEDF functional peptides inhibit prostate tumor growth—a mechanistic study. Biochem Pharmacol.

[147] Sjöström-Mattson J, Von Boguslawski K, Bengtsson NO, Mjaaland I, Salmenkivi K, Blomqvist C (2009). The expression of p53, bcl-2, bax, fas and fasL in the primary tumour and lymph node metastases of breast cancer. Acta Oncol.

[148] Li Q, Peng J, Li XH, Liu T, Liang QC, Zhang GY (2010). Clinical significance of Fas and FasL protein expression in gastric carcinoma and local lymph node tissues. World J Gastroenterol: WJG.

[149] Knechtel G, Hofmann G, Gerger A, Renner W, Langsenlehner T, Szkandera J (2010). Analysis of common germline polymorphisms as prognostic factors in patients with lymph node-positive breast cancer. J Cancer Res Clin Oncol.

[150] Seitz DH, Palmer A, Niesler U, Braumüller ST, Bauknecht S, Gebhard F (2011). Altered expression of Fas receptor on alveolar macrophages and inflammatory effects of soluble Fas ligand following blunt chest trauma. Shock..

[151] Cobbaut M, Derua R, Döppler H, Lou HJ, Vandoninck S, Storz P (2017). Differential regulation of PKD isoforms in oxidative stress conditions through phosphorylation of a conserved Tyr in the P+ 1 loop. Sci Rep.

[152] Xu J, Millard M, Ren X, Cox OT, Erdreich-Epstein A. c-Abl mediates endothelial apoptosis induced by inhibition of integrins αvβ3 and αvβ5 and by disruption of actin. Blood. 2010;115(13):2709-18. 10.1182/blood-2009-05-223776 PMC2852370 20124512

[153] Liebl J (2015). Cdk5 and Foxc2–a new relationship in the lymphatic vasculature. Oncotarget..

[154] Fu MR, Conley YP, Axelrod D, Guth AA, Yu G, Fletcher J (2016). Precision assessment of heterogeneity of lymphedema phenotype, genotypes and risk prediction. Breast.

[155] Agollah GD, Gonzalez-Garay ML, Rasmussen JC, Tan IC, Aldrich MB, Darne C (2014). Evidence for SH2 domain-containing 5′-inositol phosphatase-2 (SHIP2) contributing to a lymphatic dysfunction. PLoS One.

[156] Smpokou P, Tworog-Dube E, Kucherlapati RS, Roberts AE (2012). Medical complications, clinical findings, and educational outcomes in adults with Noonan syndrome. Am J Med Genet A.

[157] Cordeddu V, Yin JC, Gunnarsson C, Virtanen C, Drunat S, Lepri F (2015). Activating mutations affecting the Dbl homology domain of SOS2 cause Noonan syndrome. Hum Mutat.

[158] Reynaud D, Pietras E, Barry-Holson K, Mir A, Binnewies M, Jeanne M (2011). IL-6 controls leukemic multipotent progenitor cell fate and contributes to chronic myelogenous leukemia development. Cancer Cell.

